# Laparoscopy in pediatric urology: from inception to innovation - a comprehensive review

**DOI:** 10.1097/MS9.0000000000004162

**Published:** 2025-10-21

**Authors:** Sakshi Kumari, Zain Ahmed Khan, Shree Rath, Mahin Qudeer Sheikh, Farwa Fatima, Fena Mehta, Kamil Ahmad Kamil

**Affiliations:** a Department of Internal Medicine, Shimoga Institute of Medical Sciences, Shimoga, India; b Department of Surgery, Billroth Hospital, Chennai, India; c Department of Internal Medicine, All India Institute of Medical Sciences, Bhubaneswar, India; d Department of Internal Medicine, Ammer-ud-Din Medical College, Lahore General Hospital, Lahore, Pakistan; e Department of Internal Medicine, Dow University of Health Sciences, Karachi, Pakistan; f Department of Internal Medicine, Smt. NHL Municipal Medical College, Ahmedabad, India; g Internal Medicine department, Mirwais Regional Hospital, Kandahar, Afghanistan

**Keywords:** Fowler-Stephens orchiopexy, Laparoscopic ureteral reimplantation, Natural orifice transluminal endoscopic surgery

## Abstract

Laparoscopic urology has marked a significant advancement in pediatric surgery, offering less invasive procedures and improved postoperative outcomes. Initially utilized for diagnosing cryptorchidism in 1976, its therapeutic applications expanded in the 1990s to include surgeries such as orchiopexy, nephrectomy, pyeloplasty, etc. Laparoscopy, as a minimally invasive technique, allows visualization and treatment of abdominal and pelvic organs. The therapeutic potential of laparoscopy emerged in the 1990s, when Clayman and colleagues performed the first laparoscopic nephrectomy, marking the start of therapeutic laparoscopy. Since then, it has evolved significantly, becoming a standard approach in many pediatric urology centers. Technological advancements, particularly the development of miniature equipment and robotic-assisted surgery, have transformed pediatric laparoscopy, making it feasible even for neonates and infants. However, challenges persist, including increased expenses, a steep learning curve, and the need for compelling evidence before newer technologies are widely implemented. Future directions include improving telemedicine support, investigating pediatric-specific procedures, and integrating novelties such as single-site laparoscopy and natural orifice transluminal endoscopic surgery. While laparoscopic techniques have significantly advanced, further research is required to assess long-term results, cost-effectiveness, and the psychological impact on children. This narrative review highlights the revolutionary impact of laparoscopy on pediatric urology, emphasizing the need for ongoing research, training, and global collaboration to optimize outcomes and ensure wider accessibility. By addressing these challenges and refining new technologies, the future of laparoscopy in pediatric urology appears promising, with the potential to significantly improve the lives of young patients with urological conditions.

## Introduction

In pediatric surgery, where precision is crucial, laparoscopic procedures have become a highly valued advancement. Laparoscopy, as a minimally invasive technique, allows visualization and treatment of organs within the abdominal and pelvic cavities, serving both diagnostic and therapeutic purposes^[1,2]^. Its application spans multiple surgical specialties, including pediatric urology.

The first recorded use of laparoscopy in urology was in 1976, when Cortesi *et al* used it to diagnose cryptorchidism^[3]^. In the 1990s, Clayman and colleagues performed the first laparoscopic nephrectomy, marking the start of therapeutic laparoscopy^[4]^. The 1990s also saw the adoption of laparoscopic pyeloplasty, which became increasingly popular due to reduced morbidity compared to open procedures. More recently, robotic-assisted laparoscopic surgery was introduced in the early 2000s, enhancing precision in complex cases such as pyeloplasty and ureteral reimplantation, particularly in infants and toddlers. Advances like single-port laparoscopy and natural orifice transluminal surgery (NOTES) remain under evaluation but promise further reductions in invasiveness. While adoption rates are high in high-income countries, these advanced techniques face limitations in accessibility across lower-resource settings^[5]^.

**HIGHLIGHTS**

- Evolution of laparoscopic surgery with key milestones and wider applications.
- Benefits of laparoscopy in children: less pain, faster recovery, better cosmesis.
- Role of advanced tools, miniaturized instruments, and robotic-assisted surgery.
- Emerging trends: single-site laparoscopy and natural orifice endoscopic surgery.

In pediatric urology, laparoscopic procedures are associated with lower complication and mortality rates^[6]^. With experienced surgical teams and adherence to safety protocols, adverse events can be minimized. These benefits have contributed to the growing adoption of laparoscopy in pediatric care. Recently, robotic-assisted laparoscopic surgery has shown promise, especially in neonates and infants. However, more high-quality evidence is needed to validate its long-term safety and effectiveness in this population^[7,8]^.

As new technologies emerge, it is essential to evaluate the clinical impact, indications, and outcomes of pediatric laparoscopic urology. Equally important is understanding the global disparities in access to these advanced techniques. The prevalence of minimally invasive surgical methods differs significantly across regions. This narrative review explores the transformative role of laparoscopy in pediatric urologic care – highlighting both well-established procedures and innovative frontiers that are shaping the future of minimally invasive surgery in children.

The development of pediatric laparoscopic urology has been marked by several key milestones. Diagnostic laparoscopy for cryptorchidism was first performed in 1976, establishing the role of minimally invasive techniques in pediatric evaluation. The first therapeutic laparoscopic nephrectomy emerged in 1991, opening the way for more complex reconstructive surgeries. This manuscript follows the TITAN 2025 Guidelines for ethical and transparent use of AI-assisted writing tools^[9]^.

## Methodology

This article is structured as a narrative literature review aimed at synthesising recent advancements in pediatric urological laparoscopy. To compile a comprehensive and up-to-date synthesis of the role of laparoscopy in pediatric urology, a structured literature search was undertaken using the MEDLINE/PubMed database. The search was designed to capture the breadth of recent advancements and clinical experiences, and employed the Boolean search string: (“laparoscopy” OR “laparoscopic”) AND (“pediatric” OR “paediatric” OR “child” OR “adolescent” OR “infant”) AND (“urology” OR “urinary tract” OR “urologic diseases”). This ensured that a wide range of literature relating specifically to laparoscopic techniques applied within pediatric urological procedures was retrieved.

The literature search covered a publication window from January 2013 to April 2024. All retrieved publications were screened for relevance and quality, with inclusion limited to peer-reviewed studies published in English. The types of literature considered suitable for inclusion encompassed original research articles, case reports, systematic reviews, and meta-analyses. This inclusive approach allowed for both breadth and depth in capturing the evolving surgical landscape, clinical outcomes, and innovations in this domain.

A strict set of exclusion criteria was applied to ensure the validity and applicability of findings. Studies were excluded if they:

- Were published in non-English languages,
- Constituted grey literature (e.g., posters, conference abstracts, unpublished manuscripts),
- Were not peer-reviewed,
- Involved extremely small sample sizes with limited generalizability, or
- Declared conflicts of interest that could potentially compromise scientific neutrality.

Following this process, each eligible article underwent a full-text review by the authorship team. Articles were assessed based on methodological rigor, clarity of reporting, and relevance to pediatric laparoscopic urology. Emphasis was placed on capturing the evolution of techniques, indications, contraindications, complications, emerging technologies, and future directions in the field. The search was conducted on April 30, 2024. The findings from the search formed the basis of the discussion presented in the subsequent sections and have been presented as a narrative review.

## Evolution of laparoscopy in pediatric urology

The adoption of laparoscopy in pediatric urology was not an overnight phenomenon. Unlike its rapid integration in adult surgery, the integration of laparoscopic techniques into pediatric urology has taken place over decades, with significant progress made in the past 10 years towards embracing reconstructive procedures. Initially, laparoscopy encountered many challenges in pediatric settings, including a lack of appropriate devices and reluctance on the part of practitioners. However, technological advancements have played a crucial role in overcoming these hurdles^[10]^.

A crucial barrier has been the steep learning curve, demanding expertise with two-dimensional video interpretation, retroperitoneal space navigation, and non-intuitive instrument manipulation. Despite these hurdles, laparoscopy demonstrated benefits versus open surgery, including reduced pain, shorter hospital stay, and better cosmesis, fostering gradual acceptance.

Early laparoscopic instruments were bulky and not specifically designed for the smaller anatomies of children. This limited the dexterity and manoeuvrability surgeons needed for intricate procedures^[11]^. The subsequent development of miniaturised instruments specifically tailored for pediatric laparoscopy, like miniaturized pediatric telescopes (1.9 or 3.5 mm) and reusable instruments (3.5-mm trocars, scissors, and graspers), helped address these concerns^[12]^. With comparable outcomes, these advancements have greatly increased the feasibility and acceptance of laparoscopic techniques in pediatric urology.

Since open surgery success rates in children were already very high, surgeons were cautious when it came to the adoption of laparoscopy, as it meant embracing a new and unproven technique. Furthermore, a major obstacle associated with laparoscopy was the steep learning curve, which necessitated that surgeons pick up new skills and expertise such as space planning, using less intuitive movements with trocars, and deciphering a two-dimensional video image to represent a three-dimensional (3D) surgical field^[13]^. Additionally, the potential risks inherent in adopting a novel approach, particularly in young patients, underscored the need for careful consideration and thorough evaluation before widespread adoption^[10]^.

The introduction of robotic-assisted laparoscopy in pediatric urology represented a paradigm shift, providing surgeons with enhanced dexterity and visualization. Robotic-assisted laparoscopy demonstrated a shift in laparoscopic operation access from transperitoneal to retroperitoneal, and the exploration of intricate reconstructions of the gastrointestinal and urinary tract began. Laparoscopic procedures are still being developed via ongoing research and invention, and new applications like single-site laparoscopy and natural orifice transluminal endoscopic surgery show potential for future development^[14]^. However, the prevalence of such advanced techniques remains uneven, with high-income countries adopting robotic and 3D laparoscopy routinely, while LMICs continue to rely on open surgery due to limited access to resources and expertise^[15]^. Originally used as a diagnostic tool to find an impalpable testis, this technique is now widely used for complex reconstructive treatments such as pyeloplasty^[8]^.

The adoption of laparoscopic and robotic techniques in pediatric urology is markedly uneven globally. While high-income countries (HICs) demonstrate widespread access to advanced minimally invasive surgery, many low- and middle-income countries (LMICs) face systemic barriers including prohibitive costs of equipment, infrastructure deficits, lack of trained personnel, and supply chain limitations. This situation has resulted in the persistence of open surgery as the mainstay in many resource-constrained settings, impeding equitable delivery of minimally invasive care. Recent studies highlight efforts to bridge this gap through capacity-building initiatives, equipment donation programs, and tele-mentoring, though sustainable implementation remains challenging. Including LMIC perspectives enriches the discourse, urging international collaboration and technology adaptation to socioeconomic realities .

While laparoscopic surgery has been widely adopted in adult urology, its application in pediatric urology necessitates special consideration due to anatomical and physiological differences unique to children. The review expands on the significant advancements enabling procedures such as pyeloplasty, nephrectomy, and orchiopexy with minimal morbidity. However, challenges remain, including the limited working space and instrument precision required for neonates and infants. The growing integration of robotic systems has mitigated some technical challenges by enhancing dexterity and 3D visualization, although high costs and training demands restrict universal applicability.

Laparoscopic techniques in peediatric urology provide significant benefits over traditional methods, such as shortened recovery periods, shorter hospital stays, and less blood loss. In comparison to open surgery, laparoscopy is more expensive, but there are several benefits, which include reduced postoperative discomfort, less need for analgesics, and better cosmetics. The benefits of laparoscopy have been highlighted in studies comparing open and laparoscopic procedures, such as bladder augmentation and pyeloplasty. These findings reflect the growing preference in pediatric urology for minimally invasive techniques^[16,17]^.

## Indications and contraindications

In pediatric urology, laparoscopy provides a minimally invasive option for a number of diseases, but its use needs to be carefully assessed depending on particular indications and contraindications. It has a broad range of applications in pediatric urology, from diagnostic evaluations to intricate reconstructive procedures. Diagnostic laparoscopy for examination of undescended testes and laparoscopic nephrectomy have now become standard practices. Furthermore, an increasing number of benign tumors or dysfunctional kidneys are being treated with surgeries, including laparoscopic nephrectomy, partial nephrectomy, heminephrectomy, and nephroureterectomy.

Testes that are impalpable can be thoroughly examined with diagnostic laparoscopy, which also helps in evaluating the necessity for orchidectomy and the viability of single- or two-stage orchidopexy. This approach enhances visualisation and enables precise dissection, which is useful for high intra-abdominal testes. Laparoscopic orchiopexy has a success rate that is comparable to open approaches, with studies showing shorter operation times, less intraoperative bleeding, and rapid recovery time^[18]^. In a multi-institutional retrospective report, laparoscopic orchiopexy demonstrated lower rates of atrophy compared to one-stage Fowler–Stephens orchiopexy (FSO), with only 2% of testes experiencing atrophy after laparoscopic orchiopexy compared to 22% in single-stage FSO^[11]^.

Transperitoneal laparoscopic pyeloplasty offers overall success rates and complications similar to open approaches. According to earlier research, patients over the age of 10 years had the greatest benefits from laparoscopy, experiencing shorter hospital stays and less pain after surgery than younger patients. However, Chan *et al* found that after robotics was introduced, benefits also extended to younger children^[19]^. Precise intracorporeal suturing presents the biggest challenge in laparoscopic pyeloplasty. This problem is solved by robotic-assisted laparoscopic pyeloplasty, where the robotic system provides wrist-mounted instruments, allowing for a reduced learning curve and increased suturing precision^[13]^.

When a nephroureterectomy is desired, laparoscopic nephrectomy eliminates the need for a second incision by enabling extended ureteral dissection distally into the pelvis. The ureter is typically identified and traced to the renal hilum by following the psoas muscle. After that, the renal arteries are separated and individually ligated using either metal or Hem-o-Lok clips. In contrast to traditional laparoscopy, robotic-assisted laparoscopic partial nephrectomy provides enhanced visualization and dexterity, which may increase efficiency and safety, according to Deng *et al*^[20]^.

Furthermore, a multicenter study by Chan *et al* demonstrated that robotic pyeloplasty was associated with reduced hospital stay, pain scores, and comparable success rates when compared to both open and laparoscopic techniques across all pediatric age groups^[19]^. In infants, Rague *et al* reported that robotic-assisted pyeloplasty led to improved radiographic outcomes and fewer 30-day complications compared to open repair^[21]^.

Robotic-assisted laparoscopic ureteral reimplantation (LUR) is a viable treatment option for reflux correction because it reduces operating time and makes dissection and intracorporeal suturing easier. However, there are mixed results suggesting that, on average, robotic-assisted laparoscopic ureteral reimplantation in children may be inferior to open reimplant^[22]^.

Ureteropelvic junction obstruction (UPJO) can be effectively treated with technically demanding operations such as laparoscopic pyeloplasty, with results similar to open surgery, especially in children with recurrent UPJO^[23]^. Pediatric patients can now safely undergo laparoscopic adrenalectomy, a procedure that was first devised for adults^[24]^.

Strict standards are necessary for pediatric urological laparoscopy in order to guarantee patient safety and reduce hazards. Physiological effects, such as CO_2_ insufflation and postural adjustments, must be considered, particularly in individuals with anaemia or cardiac diseases. Because pneumoperitoneum can worsen hemodynamic status, patients with unstable hemodynamics might not be appropriate candidates. Fixing coagulopathy parameters is essential to prevent hemorrhage during laparoscopy^[25]^. Large abdominal adhesions from previous procedures can make conversion to open surgery necessary and raise the risk of damage. A major concern after trocar insertion is bladder injury, especially in individuals who have had prior abdominal procedures or adhesions^[26]^. Due to space constraints and equipment mobility, large tumor sizes present difficulties for laparoscopic excision and may compromise oncological results. Surgical planning is influenced by patient size and age and guided by preoperative imaging. Although there may be technical difficulties, laparoscopy has been shown to be beneficial for patients who are morbidly obese^[27]^. Because of the potential for bacterial spread and septicemia, infectious diseases such as peritonitis or abscess formation are strictly contraindicated. Precise patient evaluation and tailored surgical planning are necessary to maximize results in peediatric urological laparoscopy^[28]^. The various indications and contraindications of laparoscopy use in pediatric urology are given in Table 1.

A comparative summary of success rates, costs, and techniques for key pediatric urological procedures is presented in Table 2. Cost estimates are adapted from cost-comparison studies on pediatric pyeloplasty and ureteral reimplantation at U.S. academic centers^[16,29]^. The orchiopexy cost range is approximate and illustrative; no direct citation was found for cost. Values may vary significantly depending on healthcare system and location.

**Table 2:** Comparison of pediatric urological procedures

Procedure	Open success rate	Laparoscopic success rate	Robotic success rate	Avg cost (USD)	Notes
Pyeloplasty	94–98%	90–96%	95–99%	8000–15 000	Best outcomes with robotics^[21]^
Orchiopexy	85–95%	85–96%	~95%	5000–8000	Lower atrophy with laparoscopy^[30]^
Ureteral Reimplant	98–99%	93–96%	90–95%	12 000 +	Robotic may be inferior^[29]^

**Table 1:** Indications and contraindications of of laparoscopy in pediatric urology

Indications	Contraindications
Diagnostic laparoscopy for undescended testes	Hemodynamic instability
Orchidopexy	Severe cardiopulmonary disease
Nephrectomy/Partial nephrectomy	Uncorrected coagulopathy
Retroperitoneal tumors	Significant abdominal adhesions
Pyeloplasty	Large tumor size
Adrenalectomy	
Ureterovesical reimplantation	
Varicocele repair	

## Current challenges and limitations

The complexity of laparoscopic procedures today highlights both expertise and risks. These include general anesthesia, respiratory issues, gas embolism, hypertension, hypotension, cardiovascular problems, arrhythmias, subcutaneous emphysema, nausea, vomiting, hypothermia, thermal injuries, infections, and complications like tumor seeding and bleeding^[10]^.

Although pediatric laparoscopic techniques have advanced, they may lack precision compared to open instruments and can cause accidental injuries, such as trocar implantation. Bowel injury occurs in 0.13% of cases and may progress to perforation^[22]^. In children, complications due to veress needle or trocar placement are reported, along with abdominal abscess, bowel obstruction, haemorrhage, and stoma issues, often requiring surgery under general anesthesia^[31]^.

Visualization is another limitation; laparoscopy impairs depth perception compared to open surgery^[32]^, and poor hand-eye coordination remains an issue despite training efforts^[29]^. Mastering laparoscopy requires extensive practice, with a learning curve of 20–75 cases for pediatric procedures^[33]^; laparoscopic pyeloplasty shows improved outcomes in recent years^[34]^.

Unforeseen complications or anatomical variations may necessitate conversion to open surgery. In radical nephrectomy and tumor thrombectomy, 27.1% required conversion due to perirenal adhesion, organ invasion, or inferior vena cava adhesion^[35]^. Age is a risk factor for conversion in retroperitoneal pyeloplasty, particularly in infants under 3 months^[36]^.

Laparoscopy also incurs higher costs due to specialized instruments and equipment^[37]^. In LMICs, faulty equipment, lack of training, and expenses hinder adoption^[15]^. Procedures remain inconsistent in these regions, with robotic surgery largely absent. Troller *et al* (2024) showed that infrastructural, logistical, and economic barriers limit pediatric laparoscopy^[38]^.

Prospective studies are needed to evaluate new technologies, long-term outcomes, and comparisons with other methods. Meta-analysis can generate stronger evidence for robot-assisted and single-site laparoscopy. High cost still limits access, demanding cost-reduction strategies. More studies are needed on acceptance by children and parents, psychological impact, and consent in pediatric surgery. Frameworks like the UNCRC and Gillick competence support involving children in decisions, though many parents fail to retain full risk information^[39]^.

Despite progress, awareness of implications, inconsistent research, lack of standardization, and ethical concerns continue to limit pediatric laparoscopic urology.

## Emerging trends and future direction

Laparoscopic surgery is rapidly evolving, with robotics offering enhanced dexterity, precision, ergonomics, vision, and manoeuvrability. Robotic arms nullify tremors, integrate haptic feedback, and enable high-resolution imaging^[40,41]^. Robot-assisted pyeloplasty shows better radiographic improvement and lower complications than open repair, including in infants^[21]^.

Single-port laparoscopy uses a single umbilical incision, reducing scars and aiding recovery, with improved psychological acceptance^[42]^. NOTES accesses the abdominal cavity via natural orifices, reducing incisions, adhesions, hernias, and wound infections^[43]^. It offers less pain and shorter stays, though pediatric trials are lacking^[42]^.

3D techniques shorten operation times and improve field of vision^[44,45]^. Dual-channel optic scopes and polarised glasses create depth, while 4K cameras enhance visual acuity. Mari G *et al* showed reduced blood loss and operative time with 4K systems^[46]^. These techniques proved successful in neonates and infants undergoing pyeloureteral anastomosis^[47]^.

Fluorescence imaging aids tumor resections by differentiating normal and tumor tissue; Abdelhafeez *et al* reported success with indocyanine green in pediatric renal tumors^[48]^. AR navigation overlays anatomy and real-time data, reducing risks and aiding training^[49,50]^. It also enhances teamwork in the operating room^[51]^.

AI techniques may predict complications, suggest tailored approaches, and augment robotic precision^[51,52]^. Telepresence surgery enables remote operations and consultations, particularly in emergencies, though it remains under development^[53]^.

## Conclusion

The adoption of laparoscopic and robotic techniques in pediatric urology is markedly uneven globally. While HICs demonstrate widespread access to advanced minimally invasive surgery, many LMICs face systemic barriers including prohibitive costs of equipment, infrastructure deficits, lack of trained personnel, and supply chain limitations. This situation has resulted in the persistence of open surgery as the mainstay in many resource-constrained settings, impeding equitable delivery of minimally invasive care. Recent studies highlight efforts to bridge this gap through capacity-building initiatives, equipment donation programs, and tele-mentoring, though sustainable implementation remains challenging. Including LMIC perspectives enriches the discourse, urging international collaboration and technology adaptation to socioeconomic realities.

## Data Availability

The data sets for this study can be availed by the reader through the corresponding author (drkamilahmad1@gmail.com).
